# Emotional experiences and gender roles of men with fibromyalgia syndrome: a cross-cultural qualitative study

**DOI:** 10.3389/fmed.2024.1286729

**Published:** 2024-05-20

**Authors:** Pilar Montesó-Curto, Loren Toussaint, Angela Kueny, Ilga Ruschak, Shannon Lunn, Lluís Rosselló, Carme Campoy, Stephanie Clark, Connie Luedtke, Alessandra Queiroga Gonçalves, Carina Aguilar Martín, Ann Vincent, Arya B. Mohabbat

**Affiliations:** ^1^Primary Care, Institut Català de la Salut (ICS), Tortosa, Spain; ^2^Department of Medicine, Rovira i Virgili University, Reus, Spain; ^3^Department of Psychology, Luther College, Decorah, IA, United States; ^4^Department of Nursing, Luther College, Decorah, IA, United States; ^5^Internal Medicine Unit, Sant Pau i Santa Tecla Hospital, Tarragona, Spain; ^6^Faculty and Department of Nursing, Rovira i Virgili University, Tarragona, Spain; ^7^Research Division, United Hospital Allina Health, Minneapolis, MN, United States; ^8^Santa Maria Hospital, Lleida, Spain; ^9^Faculty of Nursing and Physiotherapy, Lleida University, Lleida, Spain; ^10^Mayo Clinic, Division of General Internal Medicine, Rochester, MN, United States; ^11^Unitat de Suport a la Recerca Terres de l’Ebre, Institut Universitari d’Investigació en Atenció Primària (IDIAP) Jordi Gol, Tortosa, Spain; ^12^Unitat Docent de Medicina de Família i Comunitària Tortosa-Terres de L‘Ebre, Institut Català de la Salut, Tortosa, Spain; ^13^Unitat d’Avaluació, Direcció d’Atenció Primària Terres de l’Ebre, Institut Català de la Salut, Tortosa, Spain

**Keywords:** emotional experience, gender role, fibromyalgia syndrome, cross-cultural studies, qualitative study

## Abstract

**Introduction:**

Gender roles may impact men with fibromyalgia, causing a high number of negative emotional states and affective disorders. There are few studies that detect men’s high emotional suffering. This study examined the emotional experience of men with fibromyalgia.

**Methods:**

A qualitative cross-cultural study utilized inductive thematic analysis was performed at the Fibromyalgia and Chronic Fatigue Unit Santa Maria University Hospital in Spain, the Fibromyalgia and Chronic Fatigue Clinic at Mayo Clinic in the US, and volunteers from the Winneshiek County in the US A total of 17 participants, 10 men from Spain and 7 men from the US were included.

**Results:**

Three themes related to feelings/emotions emerged: (1) psychological level; (2) social level; and (3) physical level. Men with fibromyalgia from Spain and the US experienced many negative emotions. Men often experience negative emotions that are worsened by common misunderstandings and social biases/stigma about their condition.

**Conclusion/implications:**

A proper assessment of emotions when evaluating the global health of men with fibromyalgia as well as the provision of emotional support would improve their mental health and therefore their overall physical health. Emotional management should be incorporated into all treatment protocols for fibromyalgia, especially for men given the gender stigma. Health policies designed by legislators, policymakers, and support agencies must be accompanied by education in gender role concepts to improve the emotions of men with FMS. The mass media will be essential for the disclosure of the emotional suffering of male patients so that society might better understand them.

## 1 Introduction

Fibromyalgia syndrome (FMS) is characterized by chronic widespread pain along with fatigue, sleep difficulties, cognitive disturbances, and mood disorders. Prevalence ranges from 2 to 8% of the world population (1). Women have a high predominance of FMS cases, approximately 80–96% (2, 3). The American College of Rheumatology states that diagnosis is based on clinical evaluation and patient reports, as there are no reliable biomarkers to identify this disease (4). All this leads to frustration on the part of patients but also health professionals, family, friends, and co-workers (5). Differences between men and women in FMS prevalence may be linked to misdiagnosis or underdiagnosis in men due to the social stigma that this is a “female disease” (6). FMS is associated with a high prevalence of emotional and affective disorders (7). Patients with FMS tend to frequently experience feelings of anger that they repress, which often leads to the inability to implement strategies to regulate their emotional state (8). It is often considered atypical if a man complains about pain, compared to a woman. Hopefully, these attitudes are changing (9). Gender differences have been difficult to study because of relatively fewer men being diagnosed with FMS (10–13). There is a high prevalence of pain, fatigue, and other common FMS symptoms among women, while the frequency of neuropsychiatric symptoms is higher among men (14).

The concept of emotion has multiple definitions. The lack of an agreed upon definition is a constant source of misunderstandings and a series of debates, mostly unproductive, between different disciplines (15). A feeling can trigger an emotion or be the response to one. In everyday language, emotions and feelings are often used interchangeably (16). It is known that emotions impact the management of FMS (17), its symptomatology (18), functioning, and adjustment (19). Emotion management can suppress or reverse negative emotions and consequently improve the experience of pain (20). Furthermore, high levels of emotional intelligence are also related to better social interaction (20, 21). However, there are very few studies on the impact of emotions in men with FMS, but the few that exist detect men’s emotional discomfort. There have been several qualitative studies exploring experiences of illness due to FMS (22–26). Moreover, there are also other mixed methods and quantitative studies of men which analyze the impact of FMS and, in turn, emotions (12, 13, 27, 28). Additionally, men reported emotions of anger, depression, anxiety, post-traumatic stress syndrome, and irritability in the setting of FMS (28).

The purpose of this study is to add to our understanding of emotional experiences in men with FMS. Additionally, we seek to understand men’s emotional experiences resulting from FMS in the context of culture, and as such, we include men from Spain and United States in our study. These cultures have considerably different healthcare systems and approaches to treating FMS and these macro-causal factors may uniquely impact the emotional experience of men with FMS.

## 2 Methods

### 2.1 Design

The design of this study was qualitative and cross-cultural, including men from Spain and the United States (US). Men with FMS provided qualitative data on their emotional experiences through focus groups and in-depth interviews (29). To facilitate the understanding of the question on emotions, we asked: What feelings/emotions do you experience because of fibromyalgia?

### 2.2 Participants and settings

The sample was purposive (30) and was composed of outpatients attending FMS units of medical facilities in Spain and the US. A total of 17 participants were obtained, 10 from Spain and 7 from the US. Collaboration of these two specialized FMS centers started through the contact of two universities, one in Spain and the other in the US, for the specific purpose of cross-cultural research projects in FMS. Shared interests centered around examining FMS from a biopsychosocial perspective where cultural and gender factors, health policies developed in each country and the organization of health systems were of considered of great importance to understand this condition.

To protect participants’ identities, we used fictitious names in reporting our results. The current study was approved by the Ethics and Research Committee of the Primary Care University Institute Jordi Gol i Gurina from Barcelona, code: 4R18/223,, and the Mayo Clinic Human Subjects Review Board, code: 10429.010. Informed consent was obtained from all participants.

### 2.3 Data collection

#### 2.3.1 Spain

Participants were selected from a list provided by the Fibromyalgia and Chronic Fatigue Clinic from Lleida, Spain and were contacted by phone. The focus groups conducted in this clinic were divided into 2, 1 month apart in 2018 (May and June). They were conducted by two health professionals trained in focus groups and a third observer familiar with the study. Each group interview took about 120 minutes, was audio-recorded, and later transcribed by two of the researchers who attended these sessions.

#### 2.3.2 US

Participants were recruited from a list provided by the Fibromyalgia and Chronic Fatigue Clinic at Mayo Clinic (Rochester, USA) and were contacted by phone, and from voluntary contact in response to public regional advertisements of the study in Luther College (Decorah, USA) in August 2018. Due to logistical difficulties in coordinating appropriate times for group sessions in the US, focus groups were not conducted and instead one-to-one interviews were deemed the best option. Two men completed a joint interview, while five men completed individual interviews in 2018 (August). Two health professionals conducted the interviews at Mayo Clinic. The individual interviews lasted between 45 and 60 min, while the joint interview lasted 120 min. The interviews were audio-recorded for accuracy and later transcribed.

### 2.4 Data analysis

Qualitative data were analyzed according to the established methods of inductive thematic analysis through a reduction process to manage and classify the data (31). All recorded data were transcribed by three members of the research team and then independently reviewed by three other researchers to verify the accuracy of the data. Then, four team researchers generated codes and grouped them into categories. The categories were organized into themes with the consensus of the whole group. Biopsychosocial and gender perspectives were analyzed (32). The data were analyzed separately in each country, but the two lead researchers had ongoing conversations to ensure standard coding criteria were in place at each site.

## 3 Results

The patient’s characteristics are shown in Table 1. Regarding the emotional experience in men with FMS, three emotional themes emerged: (1) psychological level; (2) social level; and (3) physical level (Table 2).

**TABLE 1:** Participant characteristics.

Participant	Country	Age	Civil status	Occupational level	Level of education	Number of people at home	Time from onset of symptoms to diagnosis (years)	Time living with the disease (years)
1. Steven	United States	57	Married or in couple	Active worker	4-year college	Between two and four	15–20 years	15–20 year
2. Don	United States	51	Married or in couple	Active worker	Graduate or doctoral degree	Between two and four	2 years	7 years
3. Andrew	United States	57	Married or in couple	Active worker	Graduate or doctoral degree	Between two and four	8 years	30 years
4. Matthew	United States	50	Single	Permanent disability	High School	One	1	4
5. Oliver	United States	63	Married or in couple	Permanent disability	High School	Between two and four	1996 (22 years)	22 years
6. Ryan	United States	60	Married or in couple	Temporary disability	High School	Between two and four	2 years	June 2018
7. Henry	United States	53	Married or in couple	Active worker	4-year college	Between two and four	1.5 years	2 years
1. Daniel	Spain	59	Divorced or separated	Unemployed	High School	One	3	20
2. Jack	Spain	30	Divorced or separated	Active with temporary incapacity for work	High School	Between two and four	3	4
3. Samuel	Spain	46	Married or in couple	Retired or pensioner	4-year college	Between two and four	2	10
4. Alexander	Spain	45	Married or in couple	Active with temporary incapacity for work	Elementary school	Between two and four	4	5
5. Adam	Spain	60	Married or in couple	Unemployed	High School	Between two and four	22	40
6. James	Spain	55	Divorced or separated	Retired or pensioner	High School	One	6	10
7. Jordan	Spain	35	Single	Unemployed	High School	Between two and four	17	20
8. Jonathan	Spain	55	Divorced or separated	Permanent disability	4-year college	Between two and four	2	9
9. Julian	Spain	50	Married or in couple	Permanent disability	High School	Between two and four	2	12
10. Victor	Spain	53	Widower	Retired or pensioner	High School	Between two and four	13	15

**TABLE 2:** Themes, categories on feelings/emotions in men with fibromyalgia.

Theme and categories	Spain	United States	Quotations example
**1. Psychological level**			
1.1. Anger/rage	4	6	*“Sometimes, when you cannot support the pain anymore, you feel angry and pay it with the other people. People think you are crazy. My personality has changed…” (P4-Alexander Spain)*
1.2. Frustration/impotence	19	14	*“I’m divorced, I have to pay child support, I saw myself with a lot of impotence when I finished working, I went out crying every day” (P2-Jack Spain)*
			*“Um … I mean it’s embarrassing to have this sort of thing that shuts your body down so that it feels like you can’t do anything. Yeah. I guess I would say embarrassing” (P2-Don US).*
1.3. Uncertainty/fear (in the diagnoses and the disease process)	25	29	*“I am 30 years old, and I had a lot of CT scans, resonances, colonoscopy, laparoscopies…. “In the beginning, it was all just misdiagnoses, that if you have cancer, that if you have different misdiagnoses, you get depressed, anxious and psychotic” (P2-Jack Spain)*
			*“This diagnosis… at least for me is recent. It is disappointing because you kind of want a… hey you have this blood test. It means that you have this and here is the pill for it. I guess that ties into the same general theme… uncertainty. And an unknowing in will I be able to manage this or not?” (P7-Henry US)”*
1.4. Stress	10	14	*“A doctor told me that I suffered from post-traumatic stress like war soldiers and their tragedies. I did not experience a war, but my life has been like a war…” (P2-Jack Spain)*
			*“Yeah, and now I’m not working so now there are financial worries… I’ve started selling stuff to pay bills” (P4-Mattew US).*
1.5. Anxiety	13	13	*“I’m anxious because I’ve been in pain and undergoing tests for three years. At first you think it will pass but it doesn’t” (P2-Jack Spain)*
1.6. Depression/sadness/suicidal thoughts	15	19	*“If I remember the intensity of the pain… I just couldn’t do anything… [(muffled) you know. I didn’t go out with friends or anything like that… I sort of withdrew. That was part of being depressed about it. My depression started in that period. Generalized anxiety disorder started” (P3-Andrew US)*
			*“Hitting rock bottom, we’ve all hit it several times, but you always think it can’t be worse, but it is, and each time you’re going to stop further. Like you go up two stairs and back four” (P3-Samuel Spain)*
			*“I get depression because of all my hobbies I had were physical. I’m not able to do any of them. So that’s a lot of… and my relaxation was reading. I can’t concentrate to read. All my fun stuff has gone… And my social life has gone too” (P4-Matthew US)*
1.7. Futility	16	2	*“I am feeling a zero on the left. I was complaining about what I couldn’t do as a father and couldn’t do as a worker. We feel useless” (P8-Jonathan Spain)*
			*“They were recommendations based on the fibromyalgia clinic that I went to. It sounded great during that week, and they are very good about trying to encourage you to do those things. They did all that they could do, but I haven’t honestly implemented much of anything I learned there yet… although I respect everything that they suggested. In real life you can’t turn on a dime” (P7-Henry US)*
1.8. Guilt	3	n/a	”I go to the hospital 8-9 times in a month to get medication and a nurse told me not to go so many times. I feel like a burden” (Jack, Spain)
			*“My father is 81 and my father-in-law 70 and they are better than me. You thank for them, but you see that you don’t value anything” (P4-Alexander Spain)*
1.9. Acceptance	n/a	20	“Um… I try to look at everything positively. And with fibromyalgia that is just my nature… to be positive…. That is just my nature and just the type of person I am” (P3-Andrew US)
1.10. Hope	4	5	*I have never in my life experienced hopelessness. With my congestive heart failure everyone was crying, and I was like no I don’t like to sit and worry about it. [(muffled) My attitude was positive…. I’m a problem solver. I’m a project manager and all of my career I have been a problem solver, and this is a problem solving issue. So, I just don’t have that feeling of hopelessness… you know. I just put it into problem solving. That is just my nature and just the type of person I am.(P3- Andrew US)*
1.11. Relief with diagnosis	3	3	*He pulled out the book and said it’s fibromyalgia. That was back in ’98. About 1998… that time period. It was part relief now that I know what it was, I can actually deal with it… that was my mindset at the time.” (P3-Andrew US)*
1. 12. Enjoy in leisure time (have fun) and with the ordinary daily events of life	4	7	*“I enjoy nature and my dog. I go for a walk with him, and he helps me a lot with nature, with animals, with flowers and insects. I have done a course in organic farming. (P5-Adam Spain)*
			*“I ride my motorcycle. That this summer has gone way down. Now only half hour top” (P4-Matthieu US).*
**2. Social level**			
2.1. Couple/family/social misunderstanding	15	12	*“Few people understand this illness. If you tell a person you’re in pain, they think you’re faking it. They look at you from the bottom up and see you’re fine. They think it’s not true what you say” (P8-Jonathan Spain)*
			*“I think that a lot of people feel like it is just a cop out thing. You know what I mean? I mean if I could say I have MS or this disease or that disease that’s what is causing this then they would get it better” (P1-US Steven)*
2.2. Work misunderstanding	5	6	*“I was replaced and did not get disability” (P5-Adam Spain)*
			*”Discrimination yes. My last boss… She needed someone who could be there every single day at work. It came down to that they recognized that she was discriminating me for my fibromyalgia. I mean if I am working on a skyscraper… I can’t do that in a wheelchair… and that’s obvious… you know. But if I’m in a wheelchair and I am physically in pain I shouldn’t be punished for that. At least that’s what I believe” (P3-US Andrew)*
2.3. Stressful job	4	3	*“I think about retiring every single day (laughs). That is five years away and I don’t know if I can do this for five more years. I really don’t. So, I don’t know what to do really. I am in this place right now where I just have to figure something out” (P1- Steven US)*
2.4. Misunderstanding by doctors	8	10	*“They don’t know a lot about fibromyalgia. They don’t know a lot about chronic pain in general. I don’t know. I guess someone is going to have to make some recommendation or something to figure this out. (P1-Steven US)*
**3. Physical Level**			
3.1. Suffering	7	4	*“For me, it is a struggle of continued pain” (P2-Jack Spain)*
3.2. Snowball effect suffering	3	2	*“Then comes overweight, poor sleep and other symptoms. At the beginning you do not have depression or anxiety but with chronic pain, they appear… and as time passes, the pain increases too…” (P3-Samuel Spain)*

### 3.1 Theme 1: psychological level

Most of the emotions that emerged were negative in both Spanish and US men. These negative emotions included anger/rage, frustration/impotence, uncertainty, stress, anxiety, depression/sadness/suicidal thoughts, futility, and guilt. Positive emotions were acceptance, hope, relief with diagnosis, and enjoy in leisure time.

#### 3.1.1 Anger/rage

Due to the lack of understanding of the disease by the people around them and even by the health system, men with FMS experience a great deal of anger and rage.

”You continually feel impotence, anger, and social pressure, because those around you don’t understand the disease” (P3- Spain).

“Overall, I do hate it… I’ll tell you that up front. I can get mad at it for having it… so you get the emotional anger, frustration…” (P3- US).

#### 3.1.2 Frustration/impotence

Men with FMS experience frustration and feelings of impotence because some health providers seem to not understand the condition of FMS. Men have difficulty doing chores, daily life activities, and hobbies; even the simplest tasks are difficult. Men struggle in supporting their families due to the disease’s impact on employment. Moreover, men are frustrated because it took them a long time to be diagnosed and they feel they are not getting better. They are also frustrated due to the lack of a cure or effective treatment.

”You climb 2 steps and backward. Pressure, incomprehension plus impotence…” (P3- Spain).

“If you have pain or fatigue or fogginess and you knew it was for a week, then you can manage it, but if you knew it was forever… that weighs on you” (P7- US).

#### 3.1.3 Uncertainty/fear (in the diagnosis and in the disease process)

The symptoms that appear are unpredictable and often take a considerable amount of time to diagnose. Participants have undergone numerous medical examinations and diagnostic evaluations. Many believe that they may have a serious disease such as cancer. This leads to significant feelings of fear, uncertainty, anxiety, and depression. Furthermore, they are concerned about how the disease will evolve and whether they will be able to manage the disease.

“From the onset of symptoms to having the diagnosis I had to wait 13 years…” (P10- Spain).

“I guess I do have a fear if it gets worse at some point that I would become disabled or not be able to work.” (P2- US).

#### 3.1.4 Stress

Participants consistently identified job stress. Some experience very high levels of stress similar to post-traumatic stress. They describe job stress when working and if not working financial concerns.

“I did the work of 4 people before I was diagnosed with this disease, alone and working 10 h every day” (P6- Spain).

“I identify the cause of FM with other previous diseases and especially work stress” (P1- Spain).

#### 3.1.5 Anxiety

Pain and sleep difficulties lead to anxiety in men with FMS. Although some of them have learned to manage it.

“My sleep has gotten worse, I have insomnia, I wake up suddenly with anxiety, nightmares…” (P10- Spain).

“Well, I have certainly had more anxiety. Anxiety level is decent right now too” (P5- US).

#### 3.1.6 Depression/sadness/suicidal thoughts

Men reach the limit of their emotional strength due to physical pain, economic problems, healthcare mistreatment, lack of symptomatic improvement, lack of hope in a cure, suicidal thoughts in some cases, and obsessive thoughts. Loss of social life and hobbies contributes to these feelings as well. Many believe that they are too young for the physical limitations caused by FMS. In addition, the fact of thinking that the illness is for the rest of their lives also may cause them to become depressed.

“They sent me to the psychiatrist, because with so many pains, I hit bottom at the level of committing suicide, of not being able to take more or endure more” (P10- Spain).

*Asked if he experienced hope in his recovery* “Not anymore.” “I am only 57 years old. It’s depressing.” “The whole idea of living like this for the rest of my life is just more than I can take sometimes…” (P1- US).

#### 3.1.7 Futility

Men feel lots of futile because they are not able to do the same things they used to do; most of them don’t work and their body doesn’t respond as it should. Spanish men presented more feelings of futility. Feelings of futility because men are young, and they can’t do the same things as young people or the older adult/adults.

“When I went to the gym, I saw older people doing stretches that I couldn’t do” (P7- Spain).

”During the early years of being diagnosed, I didn’t have the depression and irritability and all those things that are going on right now. It’s like my body has reached its limits” (P5- US).

#### 3.1.8 Guilt

Spanish men feel guilty that their family members have to put up with them (i.e., burden). In addition, they cannot fulfill the role of parent or breadwinner and they feel bad about it. US men didn’t identify guilt as a feeling or emotion.

“We complain about not doing what has been assigned to us and we feel bad about it” (P3- Spain).

#### 3.1.9 Acceptance

Acceptance of FMS occurred in US men versus Spanish men. Spanish men didn’t identify guilt as a feeling or emotion.

“… but then you realize well… that’s not helping me. Right now, they don’t have a cure for it. I have got to deal with it” (P3- US).

#### 3.1.10 Hope

A common experience for men is to cling to hope. For some, it seems the only solution.

”The doctors burned me the starry ganglion and it took away my pain for 3 days, it’s not worth it, but there is a hope… something can be done” (P3-Samuel Spain).

”Hope and… well there is always hope…. Again, I was just given the word fibromyalgia a couple weeks ago by Dr. XXX in Rheumatology. I was told it was an autoimmune disease… rheumatoid arthritis. Now I am told it is fibromyalgia. I’m not sure about that. Obviously, I’m not a doctor. I want to learn more. I have looked at it a little on my phone. I’m not a big I.T. guy. I don’t learn well from reading” (P6- US).

#### 3.1.11 Relief with diagnosis

The positive aspects of the disease emerged more in US men. As it is a condition that remains permanent and cannot be completely cured, they believe that they have no other solution but to accept and adapt to it. Others remark that they are more positive and put the problems on aside.

When they were diagnosed, participants felt relief, and now some of them are able to cope with the disease.

“Before we knew what we had, we suffered torments. I think we went through an ordeal of numerous diagnoses before we knew what we really had” (P3- Spain).

#### 3.1.12 Enjoy in leisure time (have fun) and with the ordinary daily events of life

Men with FMS attempt to maintain their hobbies. They do well going for a walk with their dog, having contact with nature, and controlling their breathing. However, some are no longer capable of engaging in their hobbies, which leads to significant mood changes. This emotion has not been included together with the positive emotions as it concerns only leisure time.

“We know how to enjoy many more things because it gives us the energy to continue” (P3- Spain).

“I do voice work for a radio station here in Cedar Rapids…I do it about once a week. I always look forward to and have been enjoying” (P2- US).

### 3.2 Theme 2: social level

Similar codes about feelings of misunderstanding appeared in the US and Spain. Regarding work-life, Spanish men were not currently working but referred to the difficulties perceived during their working lives.

#### 3.2.1 Couple/family/social misunderstanding

Men with FMS do not believe that those around them understand the condition. As a result of their normal external appearance, participants often feel embarrassed and ashamed, or that they might be faking an illness. Another reason is the social pressure they have to do the housework if they are the ones who stay at home and the wife works.

“I do the housework and my wife Works” (P3- Spain).

“Back home a lot of people don’t know about the disease, so when I tell them what I have they are like oh what’s that? So, they don’t realize that it is mostly women” (P4- US).

#### 3.2.2 Work misunderstanding

Men have lost many jobs due to frequent illness-related absences. Colleagues mocked them because they did not believe that their suffering was real. Some felt discriminated at work.

“I have had many job losses, people laughed, they said I was cheating” (P8- Spain).

”I have had to miss work a couple of times where it was more substantial…my coworkers noticed, and my boss noticed. I feel like there were decisions made about my role at that time that wouldn’t have been made had I been there” (P2- US).

#### 3.2.3 Stressful job

Some have stopped working and others are waiting for the opportunity to leave work since they do not know how long they will be capable.

#### 3.2.4 Role reversal

“I was autonomous, I had workers under my charge. I had to quit work and since then I have been the mother and my wife the father of the house” (P3- Spain).

#### 3.2.5 Inability to diagnose appropriately

Some clinicians inaccurately believe that they understand the disease. Other clinicians actually do have a better understanding of patients with FMS. For FMS to be identified, patients go through numerous evaluations. They have the belief that clinicians doubt if the disease exists, that not much is known about it, and that further research is needed.

“I’ve been explaining to doctors for many years, but they’re not interested. (P10- Spain).

”Started with neurology. Went to cardiology. Went to rheumatology. The doctor in rheumatology… Dr. XXX was the first to give me… to put any kind of label of anything on it. Dr. XXX was the first thing and that started out as autoimmune… rheumatoid arthritis. If doctors don’t agree with it and only a certain set of doctors whether it is just rheumatologists or whatever that’s it” (P6- US).

### 3.3 Theme 3: physical level

Similar codes about feelings of physical suffering appeared in US and Spanish men. Pain is the main symptom that makes it difficult for them to perform activities. There is a snowball effect, and the symptoms are chained one after another. The pain appears, leading to decreased physical activity, weight changes, sleeping difficulties, and mood changes (“snowball effect”). For some, as time goes by, the pain increases.

#### 3.3.1 Suffering

Chronic generalized pain is the main aspect of physical suffering identified. It makes it difficult to perform physical exercise.

“Pain that penetrates to the soul” (P8- Spain).

“I have to get down to the ground a lot sometimes. I avoid it now a lot if I don’t need to” (P1- US).

#### 3.3.2 Snowball effect suffering

First, the pain comes, and then other symptoms appear, such as difficulty sleeping, being overweight, anxiety, and depression. It is like a snowball that is increasing.

”Part of this is because it’s all a big snowballing effect, right? I like to do walking if I can for exercise because I need to lose weight, but the more I do the more I hurt. Then you don’t walk, and you don’t do things because you hurt. So, then the weight comes and then you know I don’t sleep well and then that causes more things. It’s a big snowball effect where then I turn into this hot mess (laughs)”(P1- US).

## 4 Discussion

Our findings highlighted that men with FMS experienced so many negative emotions due to the changes in their physiology but also because they had to make numerous changes to adapt their lives to the onset of FMS. It’s difficult to make these adjustments. Their bodies are imprisoned by pain and other crippling symptoms, and they have been suffering from many failed treatments (25). These emotions were associated with different events due to the nature of the disease itself, psychological factors, and the cultural and social contexts in the men’s lives. Despite these different contexts in terms of emotions between the US and Spain, more similarities than differences were found. However, US men expressed more feelings of positive emotions (mostly acceptance) and enjoyment of leisure time. Spanish men showed more feelings of futility and guilt.

Overall, men feel anger, frustration, helplessness, depression, sadness, and impotence because of the difficulty in performing the tasks of daily life, at work, and in their own families. Men also felt frustration for not being the father or spouse they used to be, due to an inability to support their family (33). The more traditional expectation for men is that they develop a conservative role within the family, as the main breadwinner. The inability to do this has a direct impact on men as they are unable to fulfill this gender mandate produces negative emotions such as worthlessness, depression, sadness, and suicidal thoughts (34). They felt futility and depression due to inability to work, engage in recreational activities, or participate in basic daily activities (32). Most of the Spanish participants were not active workers while most US participants were currently working. Patients in Spain are allowed to exempt themselves from work by receiving a small pension, while men in the US describe fewer options to leave their jobs. The Spanish health and social system provide healthcare coverage and pension provision (27). Participants in our study also identified negative emotions like anger, frustration, and impotence experiences with the misunderstandings by some healthcare professionals with did not contribute to their improvement (11, 22, 23). The misunderstanding of the disease and lack of empathy on the part of family, friends, and health professionals caused emotions of anger, rage, frustration, and helplessness (11, 25). Also, men reported the lack of recognition of FMS in men has such a strong influence on men who are affected because it is not believed that men can suffer from it, as it is mostly associated with the female sex (35).

The difficulty in diagnosis creates negative emotions such as uncertainty, fear, stress, and anxiety (26), and it was clearly observed that men endure a lot of pain and fear of being seen as complainers (24). Men with FMS stated the difficulty for men to express their feelings in order not to be labeled as inferior men, as culturally, men’s complaints may challenge their socially constructed masculinity (23).

Men in Spain felt that life was futile and they felt guilty about their condition. This is because they were not able to fulfill the traditional gender role of being a breadwinner for their household. More commonly men were unemployed, retired (pensioner), or on permanent disability. The concept of “gender” refers to culturally accepted norms, attitudes, and beliefs about what it is to be male or female. In contrast to the old-school term “gender role,” (36) gender should be understood as an ongoing process and not as a fixed entity or outcome, and that it is linguistically constructed and contextually situated. Deviating from norms can be subject to negative stigmatization and, even worse, social exclusion (37). The construction of masculinity from cultural achievement, such as sport or manual labor means that gender is vulnerable if performance cannot be sustained due to illness or physical disability (38).

Psychosocial support is needed to improve the quality of life of men with FMS (25). Pain impacts a wide variety of experiences both physically and emotionally (24). When the pain was intense, they unloaded the tension with their closest relatives (25). Both depression and FMS challenged the traditional male identity and thus had a demasculinizing effect (39, 40). There are some key limitations to the study. First, the sample size was small. Second, fewer men are diagnosed with FMS, and as such, many men may not have wanted to make themselves visible in their communities.

## 5 Conclusion

The emotional experience associated with men with FMS was largely negative (anger, frustration, helplessness, uncertainty, fear, worthlessness, futility, stress, anxiety, depression, sadness, and suicidal thoughts). Spanish men had more futility and guilt than US men. In smaller amounts, there were also positive emotions such as acceptance, hope, and relief. Acceptance and enjoyment in leisure time were more present in US men. At the social level, there is incomprehension among partners, family, health professionals, employers, and coworkers. Men also described wanting to quit or escape from work. At the physical level, a generalized and unbearable sensation of pain provokes a cascade of negative emotions at the psychological and physical levels. Clinicians should also be trained in emotional management from a gender perspective in which men are significantly stigmatized due to FMS. Findings from this study might inform health policy design by legislators, policymakers, and support agencies as they work to improve the visibility of the emotional difficulties experienced by men with FMS and improve men’s social support and therapeutic relationships with professionals.

## Data availability statement

The raw data supporting the conclusions of this article will be made available by the authors, without undue reservation.

## Ethics statement

The current study was approved by the Ethics and Research Committee of the Primary Care University Institute Jordi Gol I Gurina from Barcelona, code: CEIC- P18/073, and Mayo Clinic, IRB 10429.010. The studies were conducted in accordance with the local legislation and institutional requirements. The participants provided their written informed consent to participate in this study. Written informed consent was obtained from the individual(s) for the publication of any potentially identifiable images or data included in this article.

## Author contributions

PM-C: Writing – review and editing, Writing – original draft, Visualization, Validation, Supervision, Software, Resources, Project administration, Methodology, Investigation, Funding acquisition, Formal analysis, Data curation, Conceptualization. LT: Writing – review and editing, Writing – original draft, Visualization, Validation, Supervision, Resources, Methodology, Investigation, Formal analysis, Conceptualization. AK: Supervision, Writing – review and editing, Writing – original draft, Validation, Software, Methodology, Investigation, Formal analysis, Data curation, Conceptualization. IR: Writing – original draft, Investigation, Formal analysis. SL: Writing – original draft, Project administration, Investigation, Formal analysis. LR: Writing – review and editing, Supervision, Project administration, Investigation, Funding acquisition, Conceptualization. CC: Writing – original draft, Investigation, Formal analysis. SC: Writing – review and editing, Project administration, Investigation. CL: Writing – original draft, Project administration, Investigation. AG: Writing – review and editing, Formal analysis. CA: Writing – review and editing, Writing – original draft, Project administration. AV: Writing – review and editing, Writing – original draft, Project administration, Investigation. AM: Resources, Writing – review and editing, Project administration, Investigation.
